# Biosynthesis and characterization of platinum nanoparticles using Iraqi Zahidi dates and evaluation of their biological applications

**DOI:** 10.1016/j.btre.2021.e00635

**Published:** 2021-05-18

**Authors:** Nasreen H. Ali, Ahmed Mishaal Mohammed

**Affiliations:** Department of Chemistry, College of Science, University Of Anbar, Ramadi, Iraq

**Keywords:** Green synthesis, Platinum nanoparticles, Ovarian cancer, *Pseudomonas aeruginosa*, *Streptococcus pyogenes*

## Abstract

Platinum nanoparticles (Pt NPs) were synthesized by utilizing the Zahidi dates extract by green synthesis technique. Platinum salts were successfully reduced to their corresponding Pt NPs in the presence of aqueous dates extract which considers a rich source of phytochemicals that led to the reduction of Pt^+4^ to Pt° atoms by providing electrons for these ions. Many techniques characterized the nanoparticles. TEM analysis showed that the Pt NPs were exhibited in diameters ranging from (30−45) nm. FE-SEM images display nanoparticles in spherical shapes. AFM screening shows that Platinum nanoparticles had small size distribution. XRD diffraction examination showed that the formation of Platinum nanoparticles exhibits a face centred cubic crystalline structure by spectrum comparative to the standard confirmed spectrum of Platinum particles produced in the experiments were in the shape of nanocrystals. The fourier transform infrared spectroscopy spectrum showed various peaks ranging from (400–4000) cm^−1^ used to identify the functional groups responsible for reducing and capping of Pt NPs. Cancer cells including the ovarian cancer SKO-3 cell line and Oesophageal cancer SK-GT-4 cell line were exposed to a series of prepared Platinum nanoparticle concentrations (0.00125, 0.0025, 0.005, 0.01) M, and the inhibition rate of growth in cells was measured for 72 h. The cytotoxicity screening showed that there was a highly toxic effect on the cancer cells. Gram-negative bacterial strain *Pseudomonas aeruginosa* and Gram-positive bacterial strain *Streptococcus pyogenes* were exposed to a series of concentrations from prepared Platinum nanoparticles (0.00125, 0.0025, 0.005, 0.01) M. The results exhibited significant inhibitory activity and the rate of bacterial growth inhibition increased with increasing concentration.

## Highlights

• Pt NPs synthesis using a reduction agent from Iraqi Zahidi dates.
• Pt NPs showed cytotoxicity against SKO-3 and SK-GT-4 cell lines.
• The bactericidal activity of Pt NPs showed inhibitory activity against *P. aeruginosa* and *S. pyogenes*.

## Graphical abstract

**Figure:**
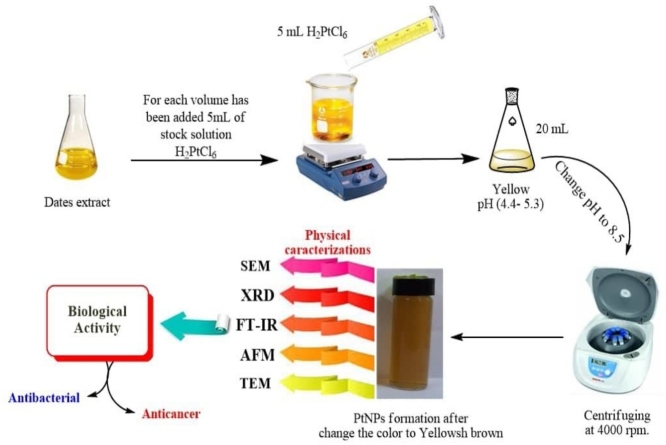

## 1 Introduction

Noble metal nanoparticles have gotten a lot of attention in recent years compared to their counter bulk materials [1]. Noble metal nanoparticles have various appealing properties, the most notable of which is their excellent catalytic performance [2]. Their unique properties, which are influenced by their size, shape, and environmental conditions, make them engaging for catalytic, sensing, anti-bacterial, and biomedical applications [3]. Nanoscale production, which is also known as nano manufacturing, is conducted by using either a bottom-up or top-down approach to the manufacture of nanomaterials, structures, devices, and systems [4,5]. The methods used for nanoparticle synthesis include the physical [6], and chemical methods [7]

Different physical and chemical processes are used to create nanoparticles. However, the physical method has several significant drawbacks, including high energy needs, complex instrumental design, high expansiveness, and poor yield. Chemical synthesis is more cost effective and produces a high yield [8]. On the other hand, these chemical methods have significant pitfalls during the fabrication stage, such as nonpolar organic solvents, hazardous substances, synthetic capping agents, and various stabilizing agents, which limits their biomedical applications [9].

In light of these conditions, green chemistry concepts are gaining popularity as a promising alternative to conventional nanoparticle synthesis approaches [10]. As a result, this technique has been proposed as an alternative to physical and chemical processes, and this alternative method has several benefits, including no toxicity, cost efficiency, accelerated synthesis, environmental friendliness, mono disperse, and large scale manufacturing, as well as eliminating of waste production and lowering production costs [11].

The green synthesis of eco-friendly metal nanoparticles from different plant derived metabolites has gained popularity in recent years and can be used as a suitable material for pharmaceutical purposes. This green nanoparticle synthesis, which has advantages over chemical processes while posing no environmental risk, assures its use in biomedical applications [12].

Green synthesis of nanoparticles is a method for producing unalloyed, morphologically superior nanoparticles in a repeatable manner using biological materials as reducing and capping agents [13].

Pt NPs are usually made using a bottom-up method, in which nanoparticles are made from Platinum precursors. Various stabilizing structures have been identified as templates for the synthesis of Pt NPs, including micelles, graphene sheets, block polymers, and microgels [14].

The water soluble organic moiety of the plants to reduce and stabilize the Platinum nanoparticles that have been prepared. Plant extracts, as compared to other traditional approaches, are more advantageous for the preparation of metal nanoparticles since they contain large amounts of biomolecules such as terpenoids, phenols, alkaloids, flavonoids, quinines, tannins, and other compounds that are responsible for the reduction and stabilization of metal nanoparticles [15].

Among these plants, date fruits are used as a mainstay food [16]. Zahidi is one of the famous types of cultivates in Iraq [17]. Date fruit has many chemical compounds with high nutritional and therapeutic qualities, and the significant constituents of dates include sugars, vitamins, amino acids, carotenoids, phenolics as flavonoids, and antioxidants [18].

Platinum nanoparticles are especially exploited for catalysis and biomedical applications [19], including pharmaceutical, manufacturing, medicinal and therapeutic applications. Pt NPs are also used in various biomedical areas, such as diagnostics with numerous imaging agents, surgical devices, drug delivery, and photo thermal therapy [20].

Several plant extracts were recently been used to produce Pt NPs. Sahin, et al. published a paper that looked at the green synthesis of monodisperse Pt NPs from *P. granatum* crusts and looked at their in vitro cytotoxic effects on the MCF-7 cell line [21]. Kumar, et al. studied the preparation of platinum nanoparticles from biologically active *Xanthium Strumarium* plant leaves, and their biological implications [22].

Cancer is the most deadly disease and there are many types of it. Ovarian cancer is the second most predominant malignancy in women over the age of 40 after breast cancer. SKO-3 human Ovarian cancer cell line is highly resistant to many cytotoxic agents [23]. Meanwhile, Oesophageal cancer is one of the most commonly recorded malignancies and the main cause of death from cancer [24]. SK-GT-4 is an Oesophageal adenocarcinoma cell line [25]. Drugs, such as cisplatin, are not selective for cancer cells, and mammalian cells are also affected. Therefore, Platinum nanoparticles manufactured by the greenway are a safe choice due to their low toxicity and imaging properties [26].

Gram-negative *Pseudomonas aeruginosa* (*P. aeruginosa*) [27]. Its capability to infect human beings with damaged natural defences and induce severe lung disease [28], *Streptococcus pyogenes* (group A beta-haemolytic Streptococcus) Gram-positive type that can be borne asymptomatically in the pharynx, skin, vagina, and rectum [29]. The therapeutic choices for treating infections are increasingly weak because of antibacterial resistance. The production of metal nanoparticles with antibacterial activity is an additional alternative to combat infections caused by antibiotic resistant bacteria [30]. Pt NPs are considered to be an alternative approach to overcome the challenges presented by multidrug resistance [31].

We aimed in this study to prepare Pt NPs by using the green method, which utilizes the Iraqi Zahidi date extract, and characterize produced Pt NPs and investigation of its effectiveness against SKO-3 Ovarian and SK-GT-4 Oesophageal cancer cell lines and inhibition rate of Gram-negative *P. aeruginosa* and Gram-positive *S. pyogenes* bacteria.

## 2 Materials and methods

### 2.1 Materials

Hydrated hexachloroplatinic [H_2_PtCl_6_.6(H_2_O)] was used as a chemical in the experiment. This chemical was purchased from Sigma-Aldrich and is used to prepare 0.01 M by dissolving 0.051791 g in 100 mL deionised water purchased from Chem-Lab (Belgium). Al-Zahidi dates were obtained from a farm in Fallujah, Anbar, Iraq, in November. A total of 0.1 M NaOH was obtained from an analytical chemistry lab at the College of Science, University of Anbar. SKO-3 and SK-GT-4 cancer cells are obtained from the cancer research centre in Baghdad. Trypsin-EDTA, RPMI-1640 supplemented with 10 % foetal bovine serum, 100 units/mL of penicillin, and 100 μg/mL of streptomycin are purchased from Capricorn (Germany). MTT(3-(4,5-Dimethyl-2-thiazolyl)-2,5-diphenyl-2H-tetrazolium bromide) was purchased from Bio World (USA). Dimethyl sulphoxide (DMSO) was obtained from Santa Cruz Biotech (USA). Muller-Hinton ((M-H)) agar was purchased from Hi-Media (India).

### 2.2 Preparation method

#### 2.2.1 Prepare dates

Zahidi dates obtained from the farm were cleaned of impurities, washed with deionised water, and dried in an oven with hot air at a temperature of 90 °C.

#### 2.2.2 Preparation Zahidi dates extract

**Fig. 1:**
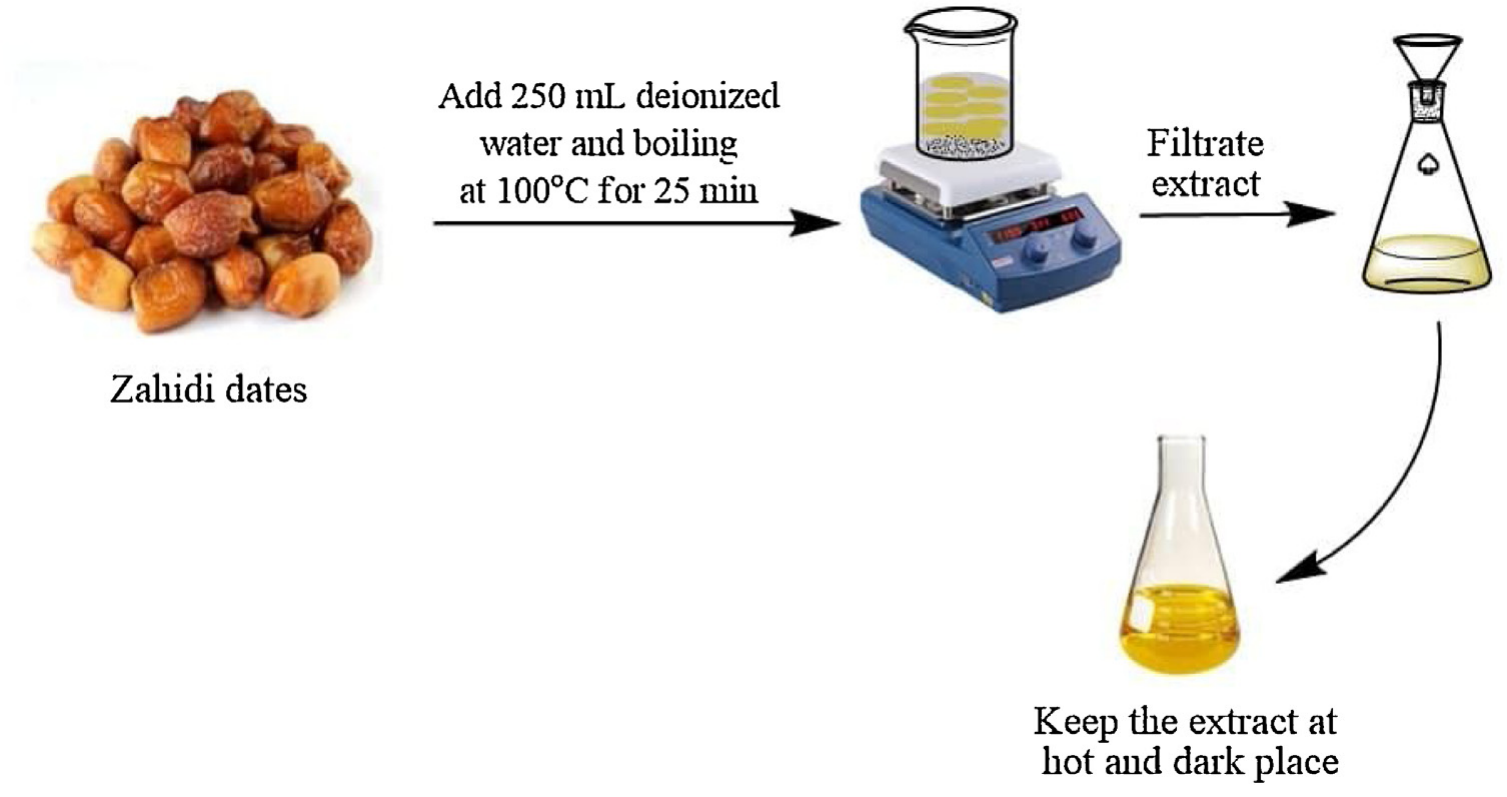
Preparation of Zahidi dates extract.

A total of 50.5 g of Al-Zahidi dates are weighed to prepare the date extract, and 250 mL of deionised water was added as a solvent. The amount was decreased to 150 mL after boiling at a temperature of 100 °C for 25 min. The solution was filtered using Whatman filter paper No. 1 to produce the filtrate (extract), which was kept in a hot and dark position and used within a day, as shown in Fig. 1.

#### 2.2.3 Synthesis of platinum nanoparticles

**Fig. 2:**
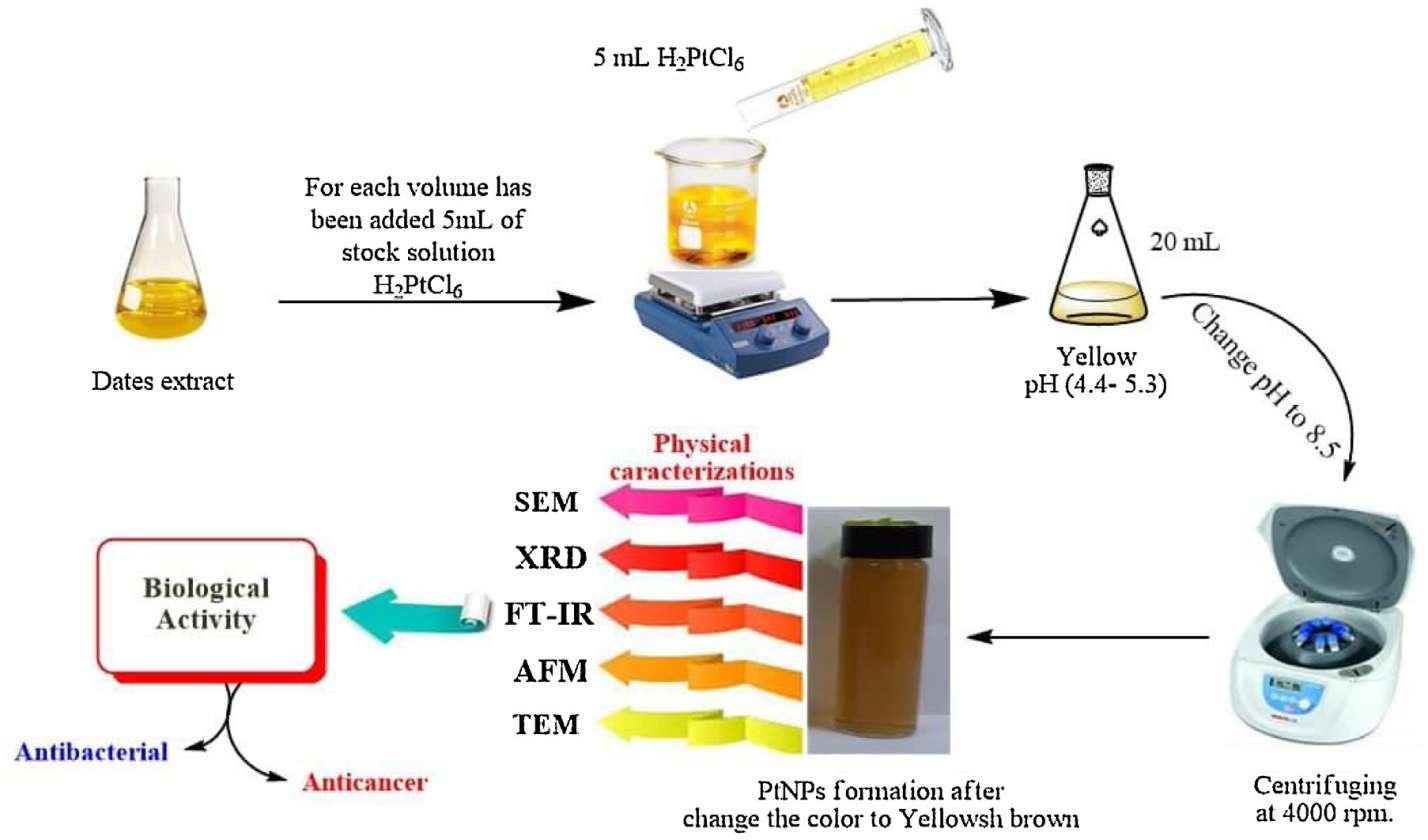
Illustration graphic for green synthesis of Platinum nanoparticles from Zahidi dates extract (For interpretation of the references to colour in this figure legend, the reader is referred to the web version of this article).

In this experiment, 6 mL of Al-Zahidi dates extract was taken and mixed with 5 mL of stock solution of [H_2_PtCl_6_.6(H_2_O)] at a temperature of 90 °C for 20 min to prepare Pt NPs. Deionised water was added to complete the volume to 20 mL, and then the solution pH was changed to 8.5 using 0.1 M NaOH. The solution changed its color from yellow to brown or yellowish-brown based on variables, such as temperature, pH, concentration, and time, as shown in Fig. 2.

#### 2.2.4 Pt NPs formation mechanism

**Fig. 3:**
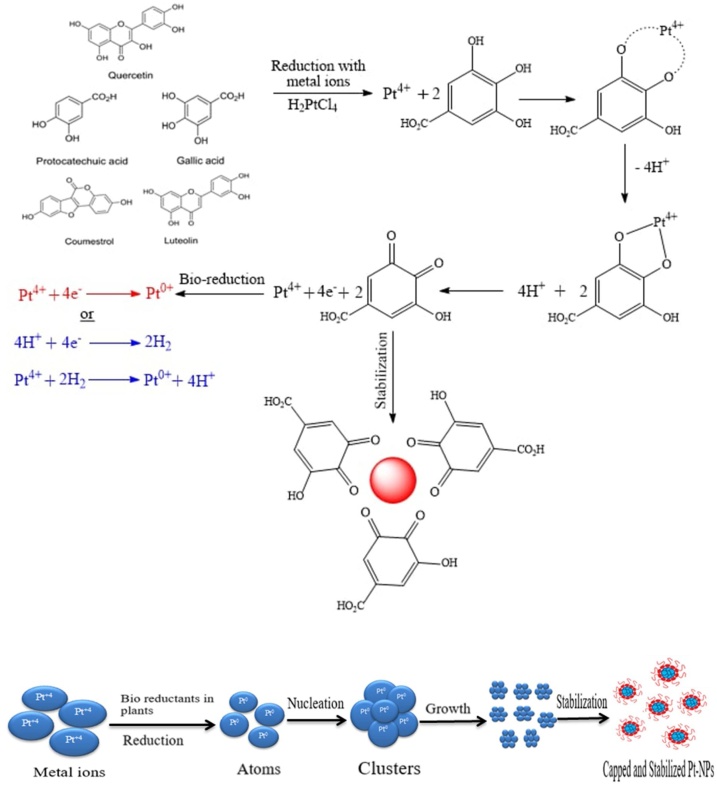
Proposed mechanism for bio fabrication and stabilization of Pt NPs by extract of plants.

In plant based sources, the bioactive compounds (e.g. polyphenols) have the dual role of a reductant and a capping agent for preserving the stabilization of metal nanoparticles [13]. The functional groups present in the Zahidi dates extract compound are thought to be the source of reducing and surface capping agents stabilizing the Pt NPs. The hydroxyl functional groups in the polyphenols will reduce Pt ions to Pt NPs and cap to form stabilized Pt NPs. The foundations of synthesis can be demonstrated in two stages. Pt atoms Pt^0^ are formed in the first step as a result of the reduction of various complexes with Pt^4+^ ions, followed by the forming of oligomeric clusters as a result of agglomeration, and these clusters ultimately contribute to the formation of colloidal Pt NPs in the second step as shown in Fig. 3. The bio reduction pathway was heavily influenced by functional groups such as amine (—NH), alcohol (—OH), carboxylic group (—COO), and amide (—CN). Groups, such as hydroxyl, are oxidized during the reduction and stabilization process, resulting in oxidized forms that start capping the surface of the Pt NPs. As a result, the functional groups in the Zahidi dates extract compounds may be responsible for the bio-reduction of Pt^+4^ ions to metallic Pt NPs as shown in Fig. 3 [32].

#### 2.2.5 Characterization of Pt NPs

Pt NPs are characterised by using the following techniques. Field emission scanning electron microscope (FE-SEM) and transmission electron microscope (TEM) (Carl Zeiss, Germany) was employed for the morphological characterisation of nanoparticles at the nanometre and nanoscale ranges. An atomic force microscope (AFM) was used to determine the dimensions of Pt NPs (ZEISS Integrated). The crystalline nature of Pt NPs was characterized by an X-ray diffraction (XRD) pattern using Philips PW1730. The solution of the reaction mixture is measured by using a UV–vis spectrophotometer (Shimadzu UV-160A). FTIR spectroscopy (IRAffinity˗1˗Shmadzo) was used to analyze the colloidal mixture of synthesized Pt NPs in the range between (400–4000) cm^−1^ to find the potential functional groups responsible for the reduction and capping agents in the Zahidi dates extract.

### 2.3 Biological applications of prepared Platinum nanoparticles

#### 2.3.1 Anticancer activity of prepared Pt NPs

##### 2.3.1.1 Culture of cells

SKO-3 and SK-GT-4 cells are maintained in RPMI-1640 supplemented with 10 % foetal bovine serum, 100 units/mL of penicillin, and 100 μg/mL of streptomycin. The cells are then passaged using Trypsin-EDTA reseeded at 80 % confluence twice a week and incubated at 37 °C [33,34].

##### 2.3.1.2 Determine cytotoxicity using MTT assay

The MTT assay is conducted using 96-well plates to determine the cytotoxic effect of Pt NPs [35,36]. Cell lines are seeded at 1 × 10^4^ cells/well. Cells are treated with tested compounds at different concentrations after 24 h or a confluent monolayer was achieved. Cells viability are measured after 72 h of treatment by removing the medium, adding 28 μL of 2 mg/mL solution of MTT, and incubating the cells for 2.5 h at 37 °C. The remaining crystals in the wells after removing the MTT solution are solubilized by the addition of 130 μL of DMSO followed by 37 °C incubation for 15 min with shaking [37]. The absorbency was determined on a microplate reader at 492 nm, and the assay is performed in triplicate. The inhibition rate of cell growth (the percentage of cytotoxicity) was calculated as follows [38,39]:

(1) Inhibition rate *=* A – B / A × 100

A is the optical density of control, and B is the optical density of the samples [40].

The cells are seeded into 24-well microtitration plates at a density of 1 × 10^5^ cells mL^−1^ and incubated for 24 h at 37 °C to visualize the shape of the cells under an inverted microscope. The cells are then exposed to PtNPs at IC50 concentration for 24 h. Afterward, the plates are stained with crystal violet stain and incubated at 37 °C for 10−15 min after the exposure time [41]. The stain is washed off gently with tap water until the dye was completely removed. The cells are finally observed under an inverted microscope at 100x magnification, and the images were captured with a digital camera attached to the microscope [42,43].

#### 2.3.2 Antibacterial activity of prepared Pt NPs

##### 2.3.2.1 Determine inhibition of prepared Pt NPs

The antibacterial behaviour of the prepared Pt NPs was examined against Gram-negative bacterial strain *P. aeruginosa* and Gram-positive bacterial strain *S. pyogenes* via agar-well diffusion technique [44]. Approximately 20 mL of (M-H) was aseptically poured into sterile Petri dishes before cultivation [33]. The bacterial species were obtained from their stock cultivars using a sterile wire loop. After the cultivation of species on the agar plates, 6 mm diameter wells were drilled using a sterile tip [45]. Various amounts of bare Pt NPs (0.01, 0.005, 0.00250, 0.00125) M were used in the bored wells. Cultivated Pt NPs containing plates and test organisms were then incubated overnight at 37 °C before measuring and recording the average diameter of the bacterial inhibition zones formed by the respective Pt NPs concentrations. The experiments were conducted in triplicate [46].

## 3 Results and discussion

### 3.1 Identification of prepared platinum nanoparticles

#### 3.1.1 TEM

**Fig. 4:**
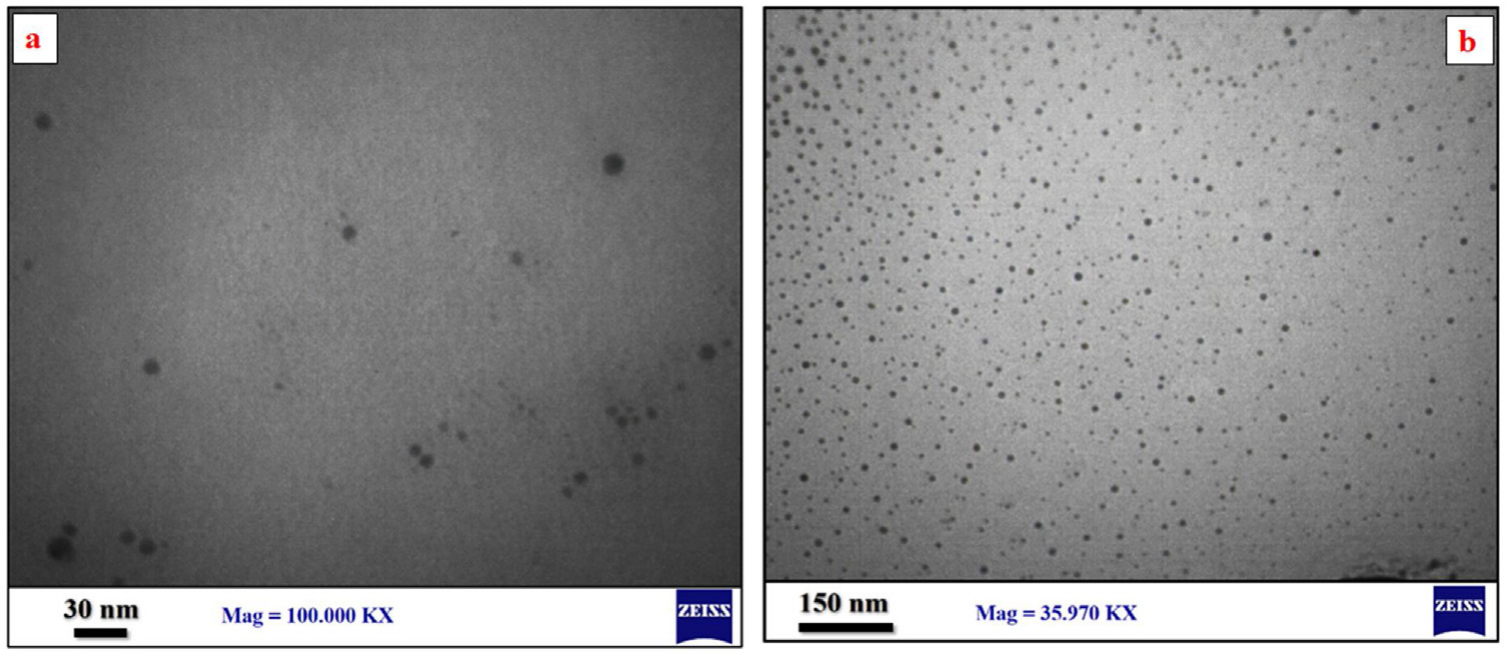
TEM micrograph Pt NPs **(a)** 30 nm and **(b)** 150 nm.

TEM analysis was recorded to study the morphological characterisation and determine the size and structure of Pt NPs. The images show that most Pt NPs have spherical shapes, as shown in Fig. 4.

The nanoparticles are arranged roughly parallel to each other, and the number of small-size number nanoparticles is remarkably high. In addition, the synthesized Pt NPs are formed in large quantities. The Pt NPs were exhibited in diameters ranging from 30 nm to 45 nm, consistent with the X-ray result.

#### 3.1.2 SEM

**Fig. 5:**
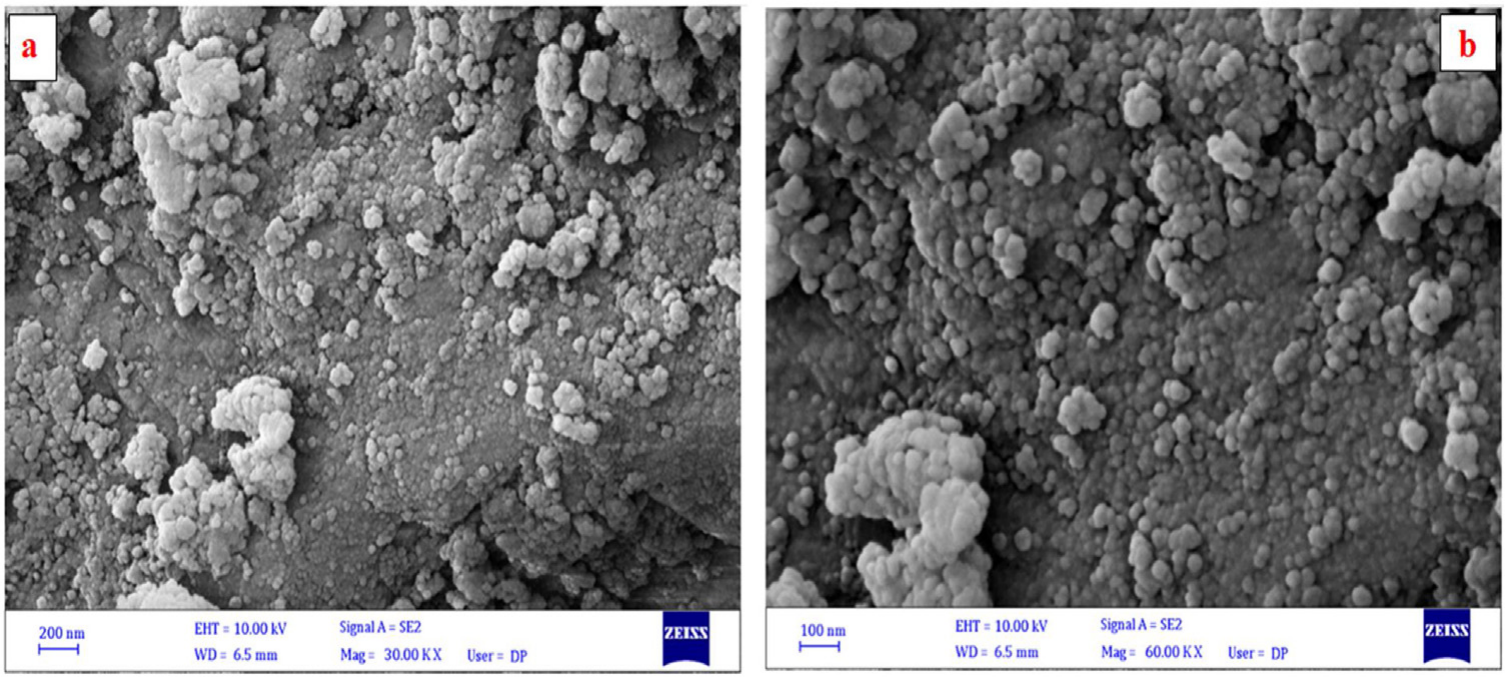
SEM micrograph Pt NPs **(a)** 200 nm and **(b)** 100 nm.

SEM analysis was accomplished to determine the shape and surface morphology of Pt NPs prepared using aqueous Zahidi dates extract with diameters ranging from 30 nm to 45 nm as demonstrated in the SEM images presented in Fig. 5.

#### 3.1.3 AFM

**Fig. 6:**
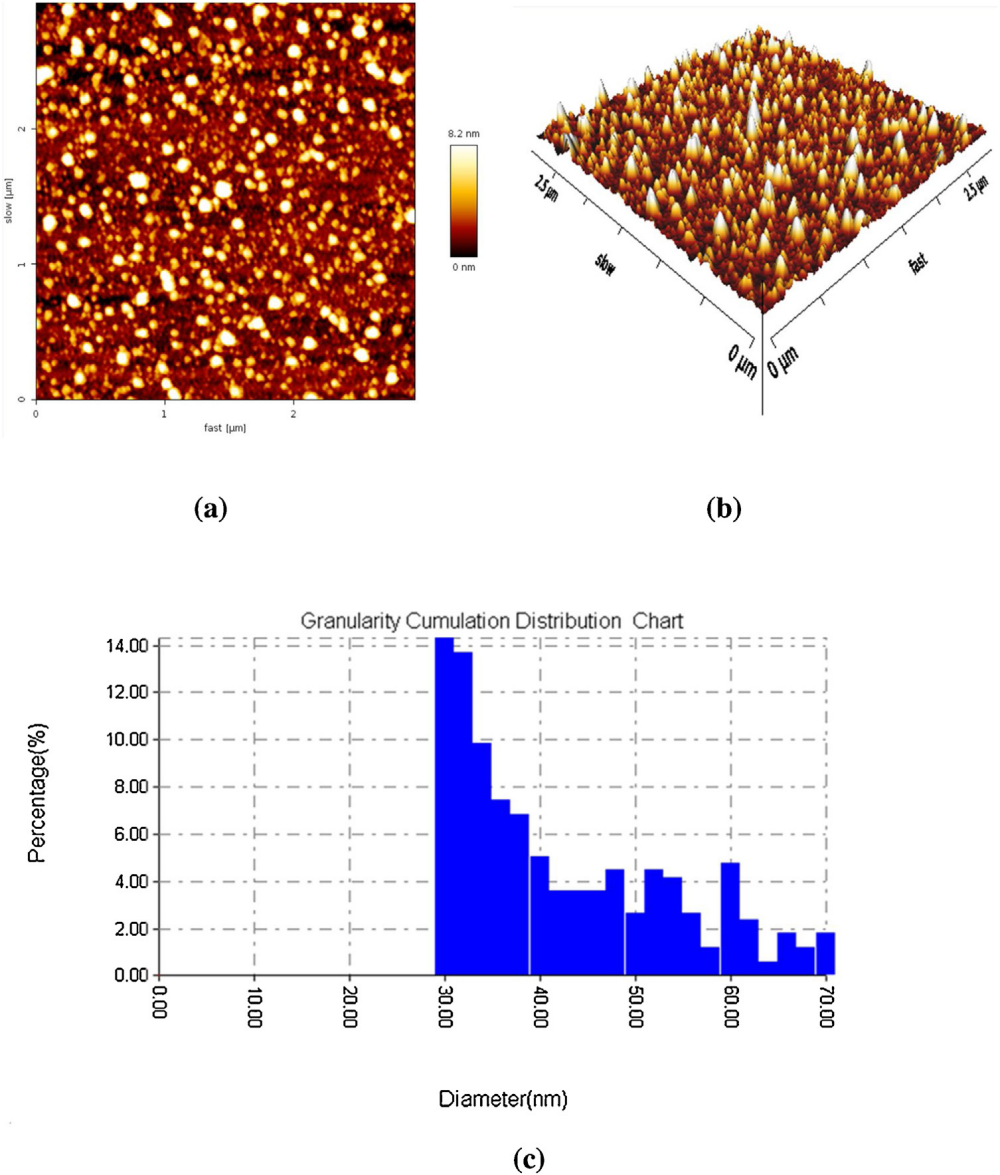
AFM analysis for Pt NPs **(a)** 2-dimension **(b)** 3-dimension structure and **(c)** Average distribution for Pt NPs.

The characterization of Pt NPs was also checked via AFM, as shown in Fig. 6. The figure displays the two- and three-dimensional images of synthesized Pt NPs. The images revealed that the distribution of Pt NPs with a diameter of 30−45 nm was limited.

#### 3.1.4 XRD

**Fig. 7:**
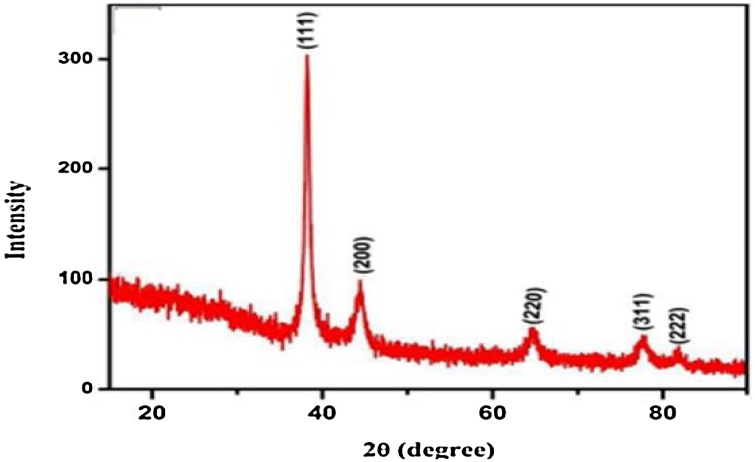
XRD of Pt NPs synthesized by Zahidi dates extract.

Pt NPs synthesized using Zahidi dates extract are crystalline. Pt NPs reveal a face-centred cubic structure. The XRD spectra of Pt NPs correspond to diffraction peaks of (111), (200), (220), (311) and (222), which were respectively assigned at angles 38.48°, 44.77°, 65.20°, 77.95° and 82.26° [47], as shown in Fig. 7.

Calculating the crystal size (D) can be done by using the Debye Scherrer equation [48]:

(2) D = Kλ/β cos θ

The average particle size of the fabricated Pt NPs was determined using the Debye-Scherrer equation, which expounds the relationship between crystallite size peak broadening in XRD in which D is the mean diameter of nanoparticles, K is the Scherrer constant with a value of 0.9, λ is the wavelength of the X-ray radiation source 0.15406 nm, and θ is the Bragg's angle [13] (Table 1).

**Table 1:** Structural parameter of Pt NPs.

No.	Pos. [°2Th.]	FWHM Left [°2Th.]	d-spacing	Height [cts]	Rel. Int. [%]	Crystallite Size [Ǻ]	Crystallite Size [nm]	Micro Strain only [%]
1	38.4648	0.1036	2.33849	123.44	100	820.444	82.0444	0.089225
2	44.4293	0.1145	2.03741	58.43	47.34	932.165	93.2165	0.109284
3	65.1921	0.09	1.42989	17.47	14.16	1266.63	126.663	0.021886
4	77.83(3)	0.036	1.22633	57(512)	46.26	1589.54	158.954	0.001709

#### 3.1.5 FT-IR

**Fig. 8:**
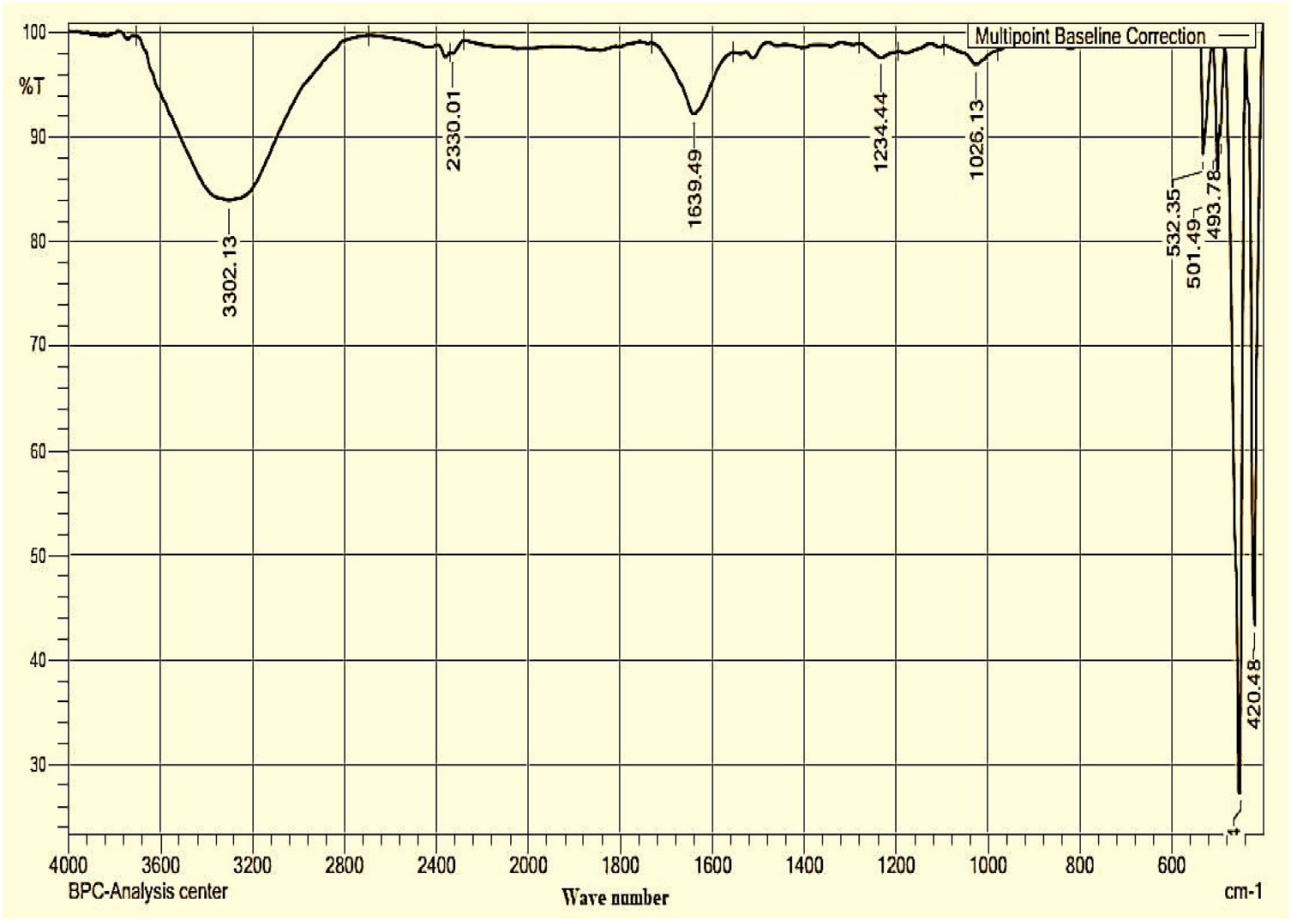
FT-IR spectrum of Pt NPs.

FT-IR spectroscopic analysis was applied to observe the possible biomolecules present in the extract of the dates, such as proteins and flavonoids. These biomolecules work as a reducing source and stabilising agent to Pt NPs synthesized by Zahidi dates extract. FT-IR spectra of Pt NPs in Fig. 8 demonstrate peaks at 3302.13, 1639.49, 1243.44, and 1026.13 cm^−1^. The broad peak at 3302.13 cm^−1^ is associated with OH stretching vibration of phenolic compounds, whilst that at 1639.49 cm^−1^ is associated with C=O stretching vibrations. The two peaks at 1243.44 and 1026 cm^−1^ are associated with the stretching vibration of C – O and C – N stretching. The FT-IR spectrum of synthesized Pt NPs illustrated a decrease in the peak intensities of the functional groups usually found in the extract of the date. The absence of these functional groups in the synthesized Pt NPs indicates the formation of Pt NPs. Sharp peaks observed in the range of 420–532 cm^−1^ refer to the vibration of PtNPs. This result indicates the successful formation of Pt NPs [49].

### 3.2 Anticancer activity

**Fig. 9:**
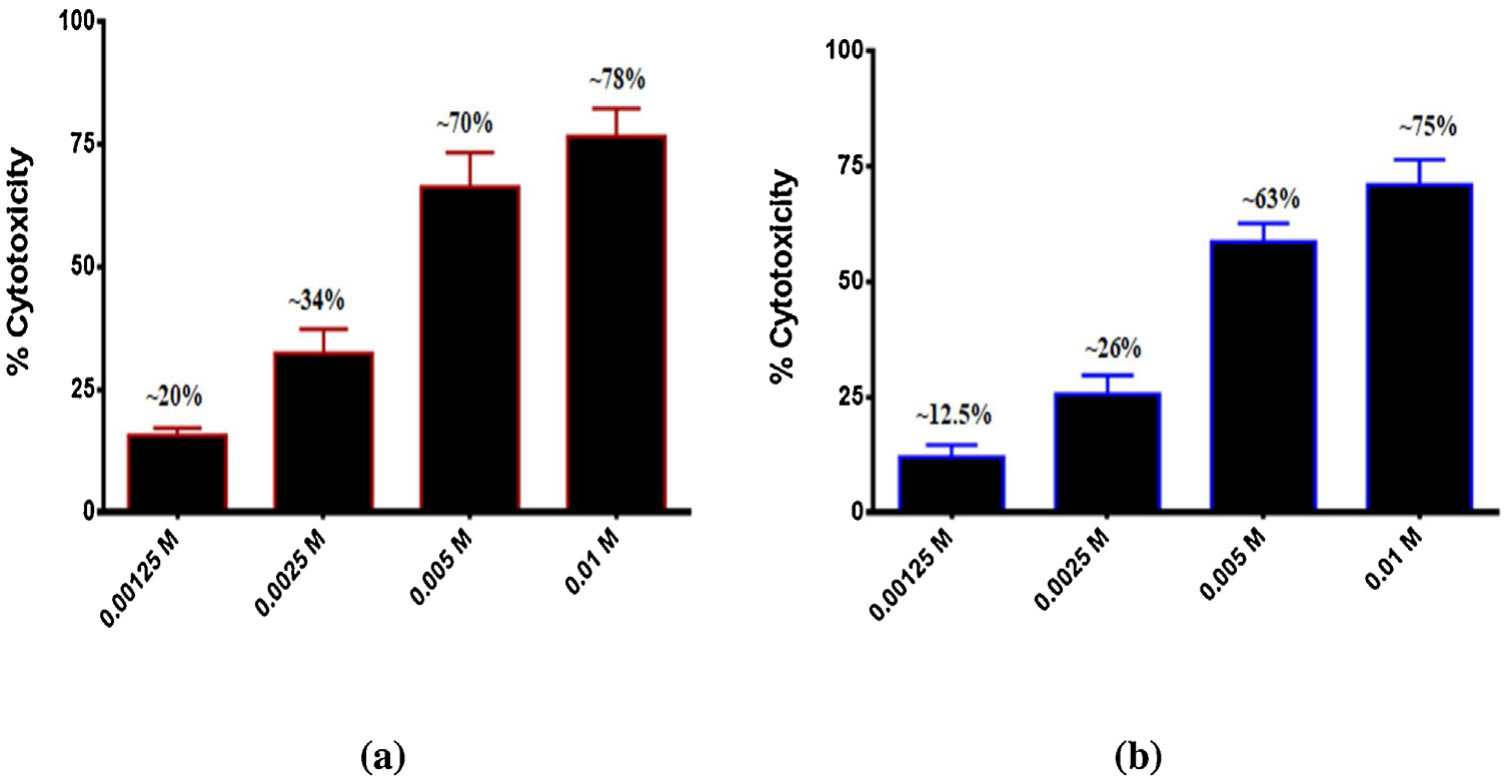
**(a)** Cytotoxic effect of Zahidi Pt NPs in SK-GT-4. **(b)** Cytotoxic effect of Zahidi Pt NPs in SKO-3 cell lines.

Developing anticancer therapeutic agents and overcoming the side effects of chemotherapy are the main objectives of researchers in medicinal chemistry. The Pt NPs synthesized by the extract of Zahidi dates were used to study their capability against cancer cells in humans [50]. Anticancer activities are conducted through the MTT method for 72 h in this study using the abnormal cell lines of SKO-3 Ovarian cancer cell line and SK-GT-4 Oesophageal cancer cell line. To study Pt NPs influences against the cancer cell lines SKO-3 and SK-GT-4. The human SKO-3 Ovarian cancer cell line and SK-GT-4 Oesophageal cancer cell line were exposed to Pt NPs at different concentrations to determine the anti-proliferative influence. The cytotoxic effect of Pt NPs on the human cancer cell lines SKO-3 and SK-GT-4 after 72 h were examined. The results showed significant inhibition of cell proliferation in cell lines. Furthermore, the inhibition of cell proliferation was significantly increased depending on concentration, as shown in Fig. 9(a) and (b).

**Fig. 10:**
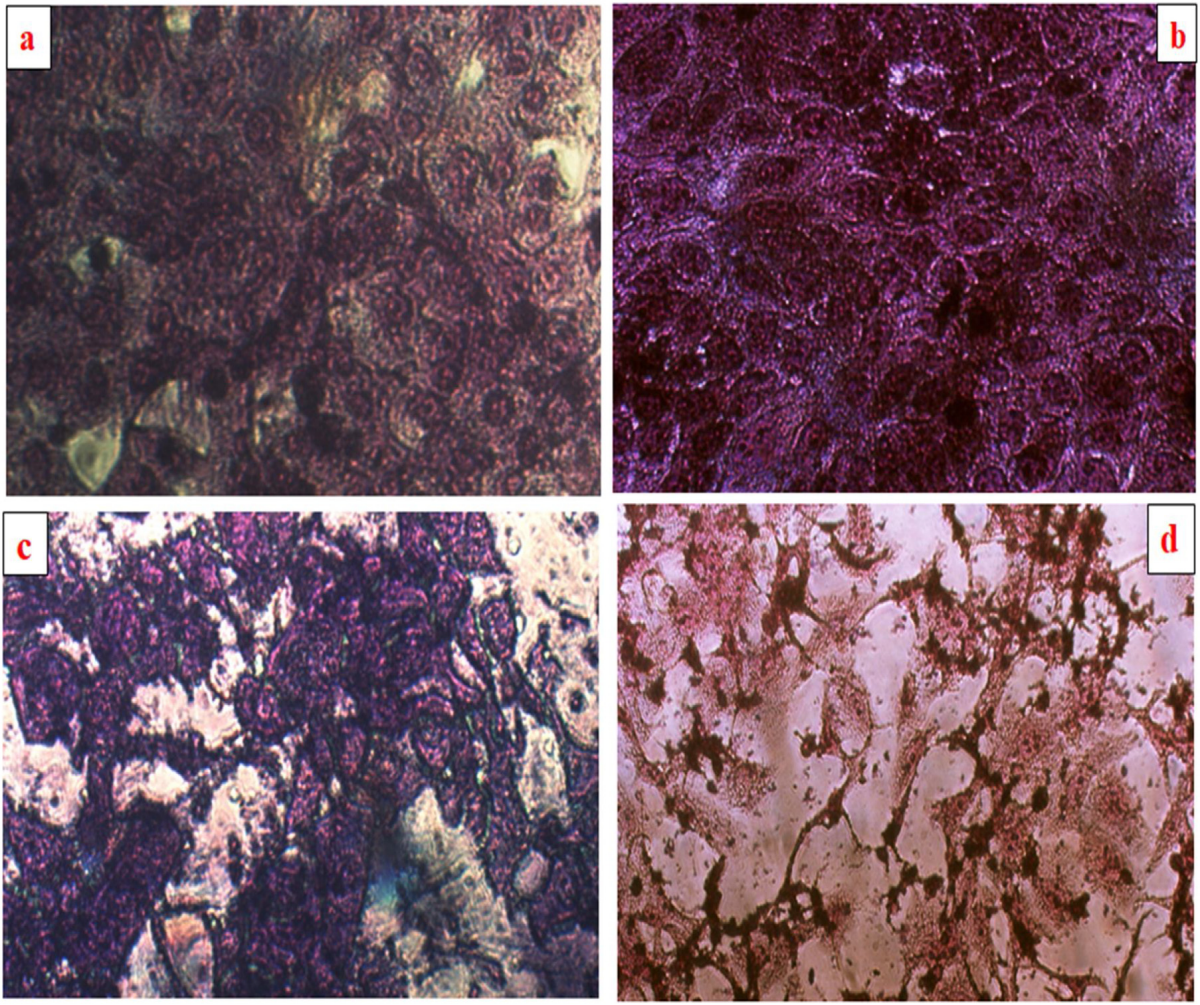
**(a)** Control untreated SK-GT-4 cells. **(b)** Control untreated SKO-3 cells. **(c)** Morphological changes in SK-GT-4 cell line after treated with Zahidi Pt NPs. **(d)** Morphological changes in SKO-3 cell line after treated with Zahidi Pt NPs.

The different concentrations of Pt NPs used in this study are as follows: (0.00125, 0.0025, 0.005, and 0.01) M. The results indicate that Pt NPs are considered to be a a precious source of effective anti-proliferative and cytotoxic substances. Bendale, *et.al* reported that Pt NPs move across cell membranes and interfere with cellular structures, implying that it has a significant effect on cell activity and viability [51]. The apoptosis property was also investigated through morphological changes in SKO-3 and SK-GT-4 cell lines. The control (untreated) cells showed that the treated cells maintained their original morphology form. By contrast, SKO-3 and SK-GT-4 cell lines treated with Pt NPs showed changes in morphology. Fig. 10(a) to (d) reveal decreased toxicity due to the reduction in the number of SKO-3 and SK-GT-4 cell colonies in those treated with the Pt NPs, thereby indicate strong cell-killing.

Alshatwi, *et.al* demonstrate that Platinum nanoparticles were discovered to have a significant cytotoxic effect on abnormal cells. As a result, these nanoparticles may be used as part of the research and development process for effective anticancer therapeutics [52].

### 3.3 Antibacterial activity

The treatment options for treating infections are rapidly restricted due to antibacterial resistance, raising the morbidity and mortality associated with infectious diseases induced by bacteria. The production of metal nanoparticles with antibacterial activity is an additional approach to combat infections caused by antibiotic-resistant bacteria [53]. Ruiz, *et.al* and Gopal, *et.al* exhibited that the antimicrobial activity of nanoparticles is based on their small size and high surface area. Nanoparticles can penetrate biofilms and bacterial cell walls, affecting intracellular processes due to their small size and large surface area. Several studies focused on the interactions for living cells with Pt NPs. The uptake and bioactivity of Pt NPs on human cells have also been examined. The Pt NPs enter the cells via diffusion and localize within the cytoplasm. DNA damage, cell aggregation, and cell apoptosis were also increased by exposure to Pt NPs [54,55].

**Fig. 11:**
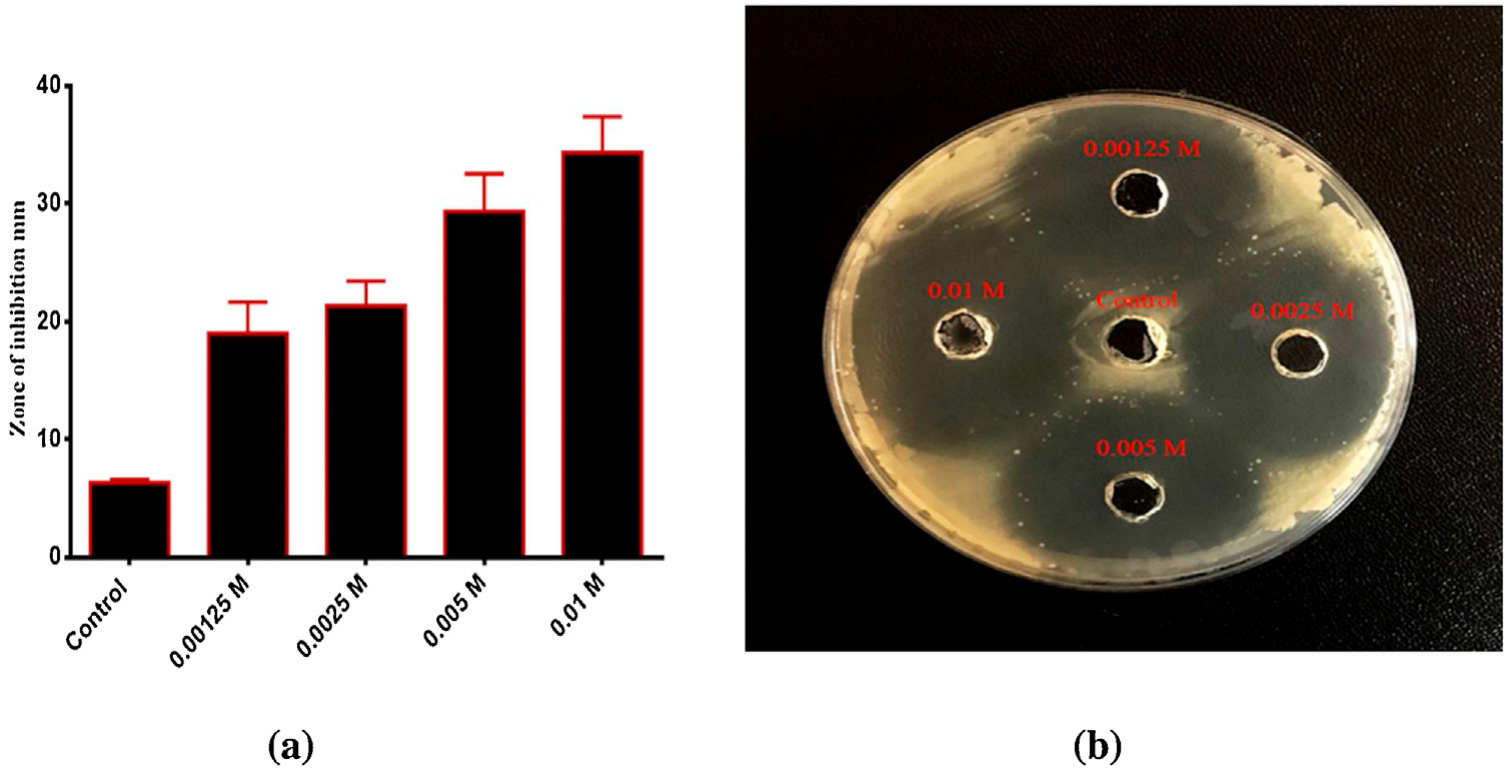
**(a)** Antibacterial activity of Zahidi Pt NPs against *Pseudomonas aeruginos*a. **(b)** Zone of inhibition for Pt NPs in mm.

**Fig. 12:**
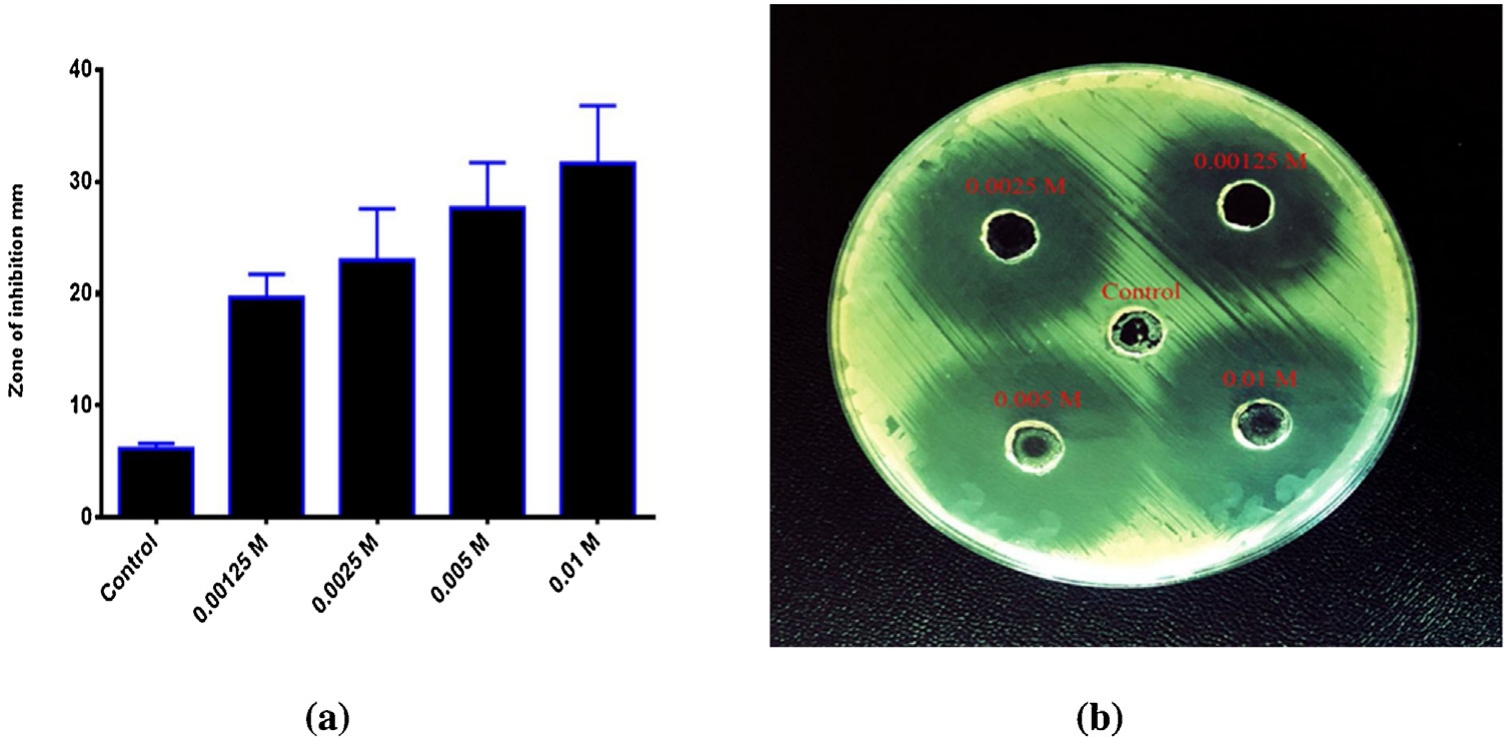
**(a)** Antibacterial activity of Zahidi Pt NPs against *Streptococcus pyogene*s. **(b)** Zone of inhibition for Pt NPs in mm.

Platinum shows vigorous activity towards many pathogenic bacteria in humans. The influence of Pt NPs as antibacterial drugs was estimated via the agar-well diffusion technique. Gram-negative bacterial strain *P. aeruginosa* and Gram-positive bacterial strain *S. pyogenes* bacteria were treated with Pt NPs in this study. Different concentrations of Pt NPs were added on Gram-negative bacterial strain *P. aeruginosa* and Gram-positive bacterial strain *S. pyogenes* which revealed inhibition zones with different diameters. The influence of Pt NPs against Gram-negative bacterial strain *P. aeruginosa* and Gram-positive bacterial strain *S. pyogenes* bacteria at different concentrations showed significant growth inhibition with an increase in dose concentration. As shown in (Fig. 11, Fig. 12), These results agree with Aygun et al. [56].

Table 2 shown inhibition areas with the diameter at various concentrations of Pt NPs as anti-bacterial against *P. aeruginosa*. In addition, Pt NPs demonstrated significant inhibition of growth with increased dose concentration.

**Table 2:** Growth inhibition of *P. aeruginosa* by Pt NPs.

Concentrations	Inhibition zone mm of *P. aeruginosa*
0.00125 M	20 mm
0.0025 M	24.5 mm
0.005 M	26.5 mm
0.05 M	32.5 mm

Table 3 shows that at varying concentrations of Pt NPs, an inhibition area with diameter was observed toward *S. pyogenes*. With increasing concentrations of Pt NPs, considerable inhibition was observed.

**Table 3:** Growth inhibition of *S. pyogenes* by Pt NPs.

Concentrations	Inhibition zone mm of *S. pyogenes*
0.00125 M	18.2 mm
0.0025 M	23 mm
0.005 M	28.4 mm
0.05 M	35.5 mm

## 4 Conclusion

Zahidi dates extract was used for the green synthesis of Pt NPs. The components of these dates worked as capping and reducing agents. The size of the obtained Pt NPs was small, and spherical shapes were observed under the preparation conditions. The PtNPs worked to inhibit the growth of Gram-negative bacteria *P. aeruginosa* and Gram-positive bacteria *S. pyogenes*. In addition, promising results obtained against Ovarian cancer human SKO-3 and Oesophageal adenocarcinoma SK-GT-4 cell lines were accomplished by Pt NPs synthesized via extracted Zahidi dates.

## Declaration of Competing Interest

The authors have declared no conflict of interest.
